# Author Correction: Substantiating freedom from parasitic infection by combining transmission model predictions with disease surveys

**DOI:** 10.1038/s41467-018-07507-0

**Published:** 2018-11-19

**Authors:** Edwin Michael, Morgan E. Smith, Moses N. Katabarwa, Edson Byamukama, Emily Griswold, Peace Habomugisha, Thomson Lakwo, Edridah Tukahebwa, Emmanuel S. Miri, Abel Eigege, Evelyn Ngige, Thomas R. Unnasch, Frank O. Richards

**Affiliations:** 1 Department of Biological Sciences, University of Notre Dame, 46556 Notre Dame, IN USA; 2 Emory University and The Carter Center, One Copenhill, 453 Freedom Parkway, 30307 Atlanta, GA USA; 3 The Carter Center, Uganda, P.O. Box 12027, Kampala, Uganda; 4 Vector Control Division, Ministry of Health, 15 Bombo Road, P.O. Box 1661, Kampala, Uganda; 5 The Carter Center, Nigeria, 1 Jeka Kadima Street off Tudun Wada Ring Road, Jos, Nigeria; 6 Federal Ministry of Health, Federal Sceretariat, Garki-Abuja, Nigeria; 7 Global Health Infectious Disease Research, College of Public Health, University of South Florida, 33620 Tampa, FL USA

Correction to: *Nature Communications* 10.1038/s41467-018-06657-5; published online 18 October 2018

The original version of this Article contained an error in the spelling of Emily Griswold, which was incorrectly given as Emily Grisworld. This error has now been corrected in both the PDF and HTML versions of the Article.

