# Supplementary material for: Substantiating freedom from parasitic infection by combining transmission model predictions with disease surveys
Source: Nat Commun. 2018 Oct 18;9:4324. doi: 10.1038/s41467-018-06657-5 (PMC6193962; doi:10.1038/s41467-018-06657-5)
Supplement: Supplementary file 1 — Supplementary Information [file 41467_2018_6657_MOESM1_ESM.pdf]

Supplementary Information

Substantiating freedom from parasitic infection by combining transmission model predictions with disease surveys

Michael et al.

Supplementary Table 1. Results from the onchocerciasis PFFI analyses where Se=0.85 and Sp=0.95.

Focus (transmission status)	Village	Year	Probability of null hypothesis, P_0	Probability of alternative hypothesis, P_a	Confidence of freedom	Classification[a]
Mt. Elgon (interrupted)	Bubungi	2005	0.023	0.986	0.977	Y
		2011	0.002	1.000	0.998	Y
	Bunabutiti	2005	0.002	1.000	0.998	Y
		2011	0.009	0.998	0.991	Y
	Bunambatsu	2005	0.035	0.987	0.965	Y
		2011	0.006	0.999	0.994	Y
	Buriri	2005	0.003	0.999	0.997	Y
		2011	0.125	0.961	0.875	insufficient evidence
Madi Mid North (ongoing)	Andra	2004	0.814	0.241	0.186	insufficient evidence
	Madulu	2004	0.167	0.878	0.833	insufficient evidence
		2011	0.052	0.971	0.948	insufficient evidence
	Masaloa	2004	0.317	0.778	0.683	insufficient evidence
	Palaure Pacunaci	2004	0.999	0.001	0.001	N

[a] Y= free from infection, N= not free from infection

Supplementary Table 2. Description of model parameters.

Parameter	Definition (units)	Parameter Range		References
		Lymphatic Filariasis Model	Onchocerciasis Model	
λ	Number of bites per vector (per month)	[5, 15]	$=\frac{H_b}{g}$	1, 2, 3, 4, 5
H_b	Human blood index	-	[0.3, 0.99]	6, 7, 8, 9, 10
g	Period of gonotrophic cycle (months)	-	[0.067, 0.13]	8, 9, 11, 12
V/H	Ratio of number of vectors to hosts	MBR[1] / λ	MBR[1] / λ	data
H_{Lin}[2]	Threshold value used in $h(a)$ to adjust the age-dependent exposure rate (months)	[240, 360]	[12, 240]	1, 3, 13
A[2]	Coefficient describing population age distribution in $\pi(a)$	data	data	data
B[2]	Coefficient describing population age distribution in $\pi(a)$	data	data	data
ψ_1	Proportion of L3 leaving vector per bite	[0.1, 0.8]	[0.12, 0.7]	8, 9, 10, 11, 14, 15
ψ_2	Larval establishment rate[3]	[0.00003, 0.00364]	[0.02, 0.0854]	1, 2, 3, 8, 9, 10, 11, 16
c	Strength of acquired immunity	[0.015, 0.025]	[0.0001, 0.001]	1, 2, 3, 17, 18
I_C	Strength of immunosuppression[4]	[0.5, 5.5]	[0.5, 5.5]	1, 2, 3, 17, 18
S_C	Slope of immunosuppression function[5] (per worm/month)	[0.01, 0.20]	[0.1, 0.75]	1, 2, 3, 17, 18
δ	Immunity waning rate (per month)	[0.001, 0.01]	[0.00001, 0.0001]	1, 2, 3, 17, 18
μ_W	Worm mortality rate (per month)	[0.008, 0.018]	[0.0083, 0.0104]	1, 2, 3, 8, 9, 10, 11, 15, 19, 20, 21, 22
τ	Pre-patency period (months)	[1, 9]	[9, 26]	11, 15, 23, 24
k_0	Basic location parameter of negative binomial distribution used in k	[0.000036, 0.000775]	[0.00036, 0.0044]	1, 2, 3, 25, 26
k_{Lin}	Linear rate of increase in k	[0.00000024, 0.282]	[0.00000024, 0.282]	1, 2, 3, 25, 26
s	Proportion of female worms	0.5	0.5	-
α	Production rate of microfilariae per worm (per month)	[0.25, 1.5]	[0.25, 1.5]	1, 2, 3, 8, 9, 10, 11, 14, 15
γ	Microfilariae mortality rate (per month)	[0.08, 0.12]	[0.08, 0.12]	1, 3, 8, 9, 10, 11, 14, 15, 21
b	Proportion of vectors which pick up infection when biting an infected host	[0.251, 0.485]	[0.259, 0.481]	1, 3, 8, 9, 10, 11, 27
κ	Maximum level of L3 given Mf density	[3, 5]	[1.16, 2.00]	1, 3, 28, 29
r	Gradient of Mf uptake[6]	[0.04, 0.25]	[0.01,0.0495]	1, 3, 28, 29
σ	Vector mortality rate (per month)	[1.5, 8.5]	[1.5, 8.5]	1, 3, 8, 9, 10, 11, 26
σ_e	Excess vector mortality due to mf infection (per month)	-	[0.75, 4.25]	30
$\boldsymbol{\sigma_L}$	Larval mortality rate	-	[0.33, 1.16]	8, 9, 10, 11

[1]Note MBR (monthly biting rate) serves as an input to initialize the model, measured as bites per person per month, the value of which may be obtained from entomological surveys conducted in study sites. In the absence of the observed MBR value, the model has been adapted to estimate it from the community-level Mf prevalence data.
[2]The parameters A, B, and H_{Lin} are estimated from national human demographic data or from the age-prevalence data.
[3]The proportion of L3-stage larvae infecting human hosts that survive to develop into adult worms [2].
[4]The facilitated establishment rate of adult worms due to parasite-induced immunosuppression in a heavily infected human host.
[5]The initial rate of increase by which the strength of immunosuppression is achieved as W increases from 0 [18].
[6]The gradient of Mf uptake r is a measure of the initial increase in the infective L3 larvae uptake by vector as M increases from 0 [2, 13].

Supplementary Table 3. Description of model functions and functional forms.

Function	Functional form
$h(a)$: age-dependent exposure rate	$= \begin{cases} \dfrac{a}{H_{Lin}} \text{ for } a < H_{Lin} \\ 1 \text{ for } a \geq H_{Lin} \end{cases}$
k : parasite aggregation	$= k_0 + k_{Lin}\overline{M(a,t)}$
ζ : rate of pre-patent worm maturation	$= e^{-\mu\tau}$
$\phi[W(a,t),k]$:worm mating probability	$= 1 - \left(1 + \dfrac{W}{2k}\right)^{-(1+k)}$
$\pi(a)$: population age distribution	$= Ae^{(-Ba)}$
$f[M(a,t),k]$: Vector Mf uptake response	$= \begin{cases} \dfrac{2}{\left[1 + \dfrac{M(a,t)}{k}\left(1 - e^{-\frac{r}{\kappa}}\right)\right]^k} - \dfrac{1}{\left[1 + \dfrac{M(a,t)}{k}\left(1 - e^{-\frac{2r}{\kappa}}\right)\right]^k} & \text{for vectors with cibarial armature (i.e. Culex mosquitoes)} \\ \left[1 + \dfrac{M(a,t)}{k}\left(1 - e^{-\frac{r}{\kappa}}\right)\right]^{-k} & \text{for vectors without cibarial armature (i.e. Anopheles mosquitoes, } \textit{Simulium damnosum/Simulium neavei} \text{ blackflies)} \end{cases}$
Φ : larval establishment rate	$= \lambda \dfrac{V}{H} h(a)\psi_1\psi_2$
F_1 : human immunity to larval establishment	$= \dfrac{1}{1 + cI(a,t)}$
F_2 : human immunosuppression	$= \dfrac{1 + I_C S_C W_T(a,t)}{1 + S_C W_T(a,t)}$
F_3 : Mf production in the human host	$= \alpha s \phi[W(a,t),k]W(a,t)$
F_4 : L3 stage larval density in the vector	$= \begin{cases} \dfrac{\lambda\kappa b \int \pi(a)(1 - f[M(a,t),k])da}{\sigma + \lambda\psi_1} & \text{for lymphatic filariasis model} \\ \dfrac{\lambda\kappa b \int \pi(a)(1 - f[M(a,t),k])da}{\sigma_L + \sigma + \lambda\psi_1 + \sigma_e \int \pi(a)h(a)M(a,t)} & \text{for onchocerciasis model} \end{cases}$

Supplementary Methods

Modeling the effects of mass drug administration

For the Nigerian lymphatic filariasis (LF) endemic sites without baseline microfilariae (mf) prevalence data, baseline model estimates were hindcasted by defining plausible ranges of initial mf and annual biting rate conditions, simulating the observed rounds of mass drug administration (MDA), and fitting the LF model to post-intervention mf data (see Methods in main text). MDA type and coverage data were retrieved from Richards et al. [31]. The impact of annual mass drug treatment was modelled by assuming that anti-filarial treatment with various drug regimens acts by killing certain fractions of the populations of pre-patent (*P*) and patent (*W*) adult worms and mf (*M*) instantly after drug administration. We denote these fractions as ω for adult worms, and ε for mf, the values of which vary according to the drug that is administered and the targeted parasite [32]. The population sizes of worms and microfilariae after drug treatment are calculated by modifying the populations of each parasite stage obtained immediately prior to the treatment:

$$\left.\begin{aligned} P(a,t+dt) &= (1-\omega C)P(a,t) \\ W(a,t+dt) &= (1-\omega C)W(a,t) \\ M(a,t+dt) &= (1-\varepsilon C)M(a,t) \end{aligned}\right\}\text{at time } t = T_{MDAi} \qquad (1)$$

In the above, dt represents a short time-period since the time-point T_{MDA_i} when the ith MDA was administered. The parameter C is the population level drug coverage. Apart from instantaneous killing of adult worms and mf, filariasis drug regimens are also thought to reduce the production of mf by worms surviving each MDA. Here we modeled this effect by introducing a new parameter (denoted by δ_{reduc}) as follows:

$$\frac{\partial M}{\partial t} + \frac{\partial M}{\partial a} = (1-\delta_{reduc}C)s\alpha\phi[W(a,t),k]W(a,t) - \mu_2 M(a,t),\ \text{for } T_{MDAi} < t \le T_{MDAi} + P \qquad (2)$$

where $\alpha' = \alpha(1-\delta_{reduc}C)$ reflects the suppressed fecundity (over a period of T_P months since the ith MDA) of adult worms that survive the administration of drugs at each MDA.

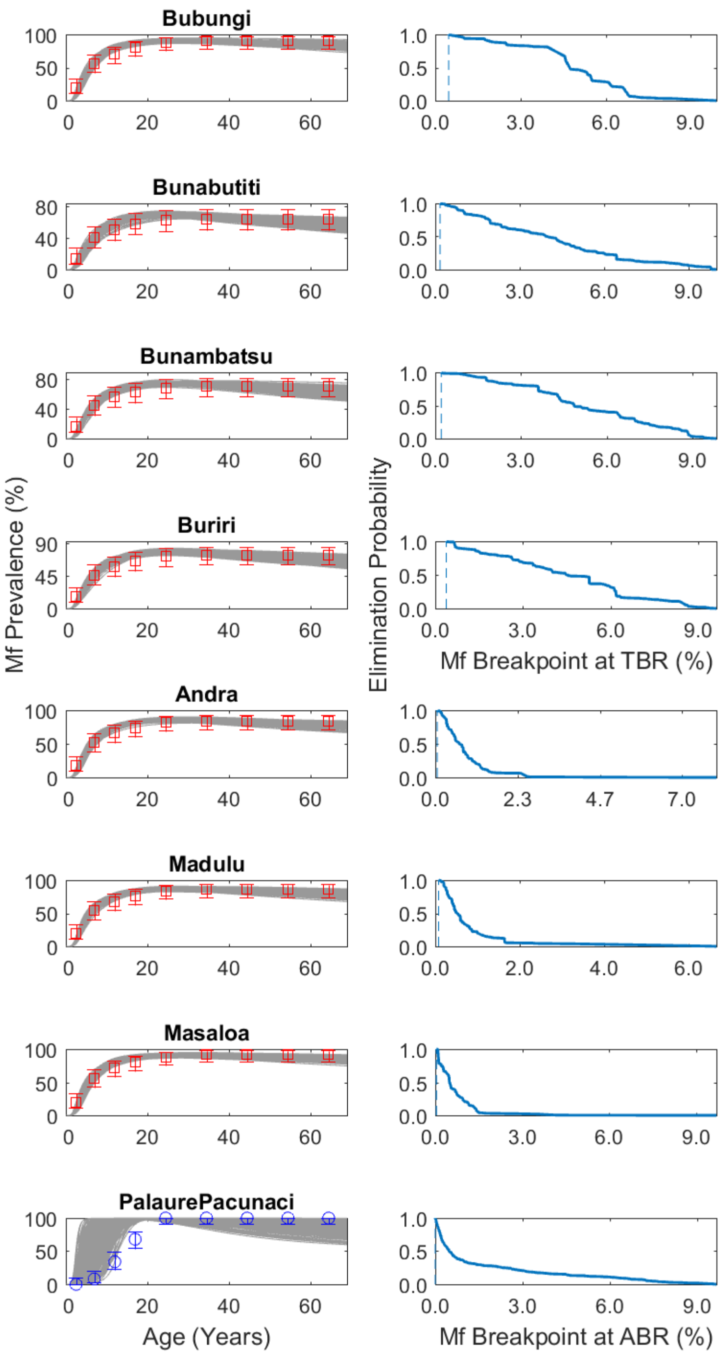

Supplementary Figure 1. Model fits and estimated transmission breakpoints for onchocerciasis endemic sites. The model fits (gray curves) to baseline microfilariae prevalence from eight onchocerciasis endemic sites are shown. Age-stratified Mf prevalence patterns (shown in the figure as red squares for plateau-type and blue circles for convex-type patterns) used for fitting were constructed according to the reported community-level Mf prevalence (Table 1). The error bars represent the 95% binomial confidence intervals. The constructed age pattern which best matched the community-level Mf prevalence based on mean-squared error calculations was used for fitting. The empirical cumulative density functions (right) of the model-calculated Mf breakpoints are shown for each site. The vertical dashed lines in the ECDF plots denotes the Mf breakpoint values corresponding to the 95% elimination probability thresholds applicable in each village. Note that the breakpoints for Bubungi, Bunabutiti, Bunambatsu, and Buriri were calculated at the threshold biting rate because vector control was used in these sites, while the breakpoints for Andra, Madulu, Masaloa, and Palaure Pacunaci were calculated at the annual biting rate.

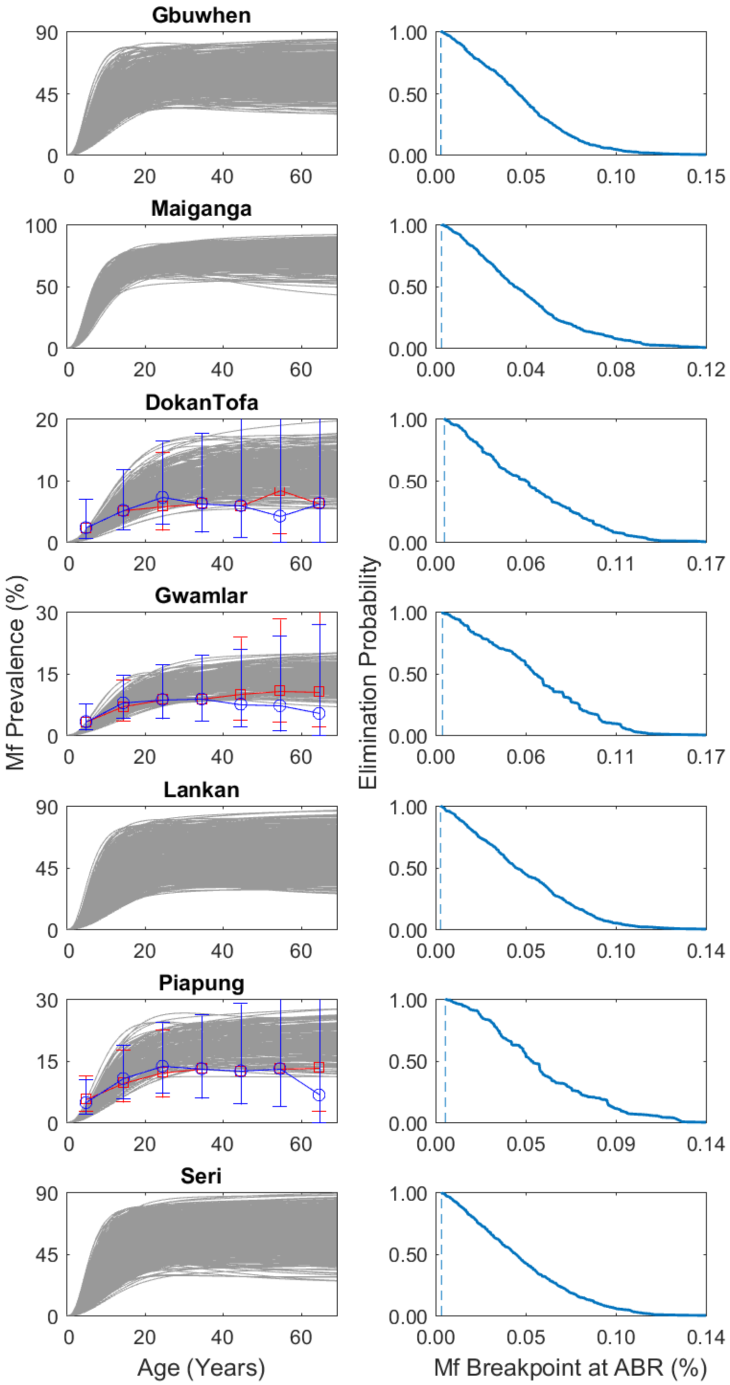

Supplementary Figure 2. Model fits and estimated transmission breakpoints for lymphatic filariasis endemic sites. The model fits (gray curves) to baseline microfilariae prevalence from seven lymphatic filariasis endemic sites are shown. Age-stratified Mf prevalence patterns (shown in the figure as red crosses for plateau-type and blue circles for convex-type patterns) used for fitting were constructed according to the reported community-level Mf prevalence (Table 2). The error bars represent the 95% binomial confidence intervals. Both plateau and convex constructed age patterns were used as an ensemble. In those sites that do not have constructed data shown, the baseline curves were hindcasted from fits to post-intervention data. The empirical cumulative density functions (right) of the model-calculated Mf breakpoints are shown for each site. The vertical dashed lines in the ECDF plots denotes the Mf breakpoint values corresponding to the 95% elimination probability thresholds applicable in each village. Breakpoint values were calculated at the annual biting rate.

Supplementary Note 1: Software

Description

The PFFI function description and code that follows allows the calculation of freedom from infection probabilities in R. PFFI analyzes data from a parasitic infection survey for determining whether a population is free from infection at the design prevalence. The calculations employ the exact hypergeometric distribution as given by Cameron and Baldock 1998, and are based on one-stage sampling, use of an imperfect diagnostic test, and a finite population.

Usage

```
PFFI(p, …)
```

Arguments

p	the design prevalence given as a proportion
Se	the sensitivity of the diagnostic test
Sp	the specificity of the diagnostic test
N	the population size
n	the number of individuals sampled in the survey
x	the number of sampled individuals who test positive for infection
alpha	the desired rate of Type I error
beta	the desired rate of Type II error

Value

A data frame is returned which contains the probability of the null hypothesis, the probability of the alternative hypothesis, the confidence of freedom, and the survey freedom classification.

References

Cameron and Baldock, (1998) A new probability formula for surveys to substantiate freedom from disease. *Preventative Veterinary Medicine*, 34(1), 1-17.

Examples

```
p = 0.005
Se = 0.8
Sp = 0.95
N = 1500
n = 200
x = 1
```

```
alpha = 0.05
beta = 0.05

out = PFFI(p,Se,Sp,N,n,x,alpha,beta)
```

Code

```
PFFI <- function(p,Se,Sp,N,n,x,alpha,beta){

# probability of null hypothesis (prob that prevalence >= design
# prevalence)

  d = floor(p*N) # diseased in population
  P_null = 0
  for (x1 in 0:x){ # summation of P(T+=x)
    c = 0
    for (y in 0:d){ # outer summation of hypergeometric
      a = (choose(d,y)*choose(N-d,n-y))/choose(N,n)
      b = 0
      for (j in 0:min(x1,y)){ # inner summation of hypergeometric
        b = b + choose(y,j)*Se^j*(1-Se)^(y-j)*choose(n-y,x1-j)*(1-
           Sp)^(x1-j)*Sp^(n-x1-y+j)
      }
      c = c + a*b
    }
    P_null = P_null+c
  }

# probability of alternative hypothesis (prob that prevalence < design
# prevalence)
  d=0 # disease free population
  P_alt = 0
  for (x2 in x:n){ # summation of P(T+=x)
    c = 0
    for (y in 0:d){ # outer summation of hypergeometric
      a = (choose(d,y)*choose(N-d,n-y))/choose(N,n)
      b = 0
      for (j in 0:min(x2,y)){ # inner summation of hypergeometric
        b = b + choose(y,j)*Se^j*(1-Se)^(y-j)*choose(n-y,x2-j)*(1-
           Sp)^(x2-j)*Sp^(n-x2-y+j)
      }
      c = c + a*b
    }
    P_alt = P_alt+c
  }

 # probability of freedom = 1-P_null
  P_free = (1-P_null)

# draw a conclusion regarding whether the survey indicates the
```

```
# population is free from infection
  if (P_null>alpha && P_alt>alpha){
    conc = "insufficient evidence, sample size too small"
  }
  else if (P_null<alpha && P_alt>(1-beta)){
    conc = "free from infection"
  }
  else if (P_alt<(1-beta)){
    conc = "not free from infection"
  }

# return list of probabilities
  vars = c('P_null','P_alt','P_free','Decision')
  Prob = c(P_null,P_alt,P_free,conc)
  out = data.frame(Prob,row.names = vars)

  return (out)

}
```

Supplementary References

1. Gambhir M, Michael E. Complex Ecological Dynamics and Eradicability of the Vector Borne Macroparasitic Disease, Lymphatic Filariasis. *PLoS One* **3**, (2008).

2. Gambhir M, *et al.* Geographic and ecologic heterogeneity in elimination thresholds for the major vector-borne helminthic disease, lymphatic filariasis. *BMC Biol* **8**, (2010).

3. Singh BK, *et al.* Sequential Modelling of the Effects of Mass Drug Treatments on Anopheline-Mediated Lymphatic Filariasis Infection in Papua New Guinea. *PLoS One* **8**, (2013).

4. Rajagopalan P. Population dynamics of Culex pipiens fatigans, the filariasis vector, in Pondicherry—Influence of climate and environment. *P Indian Acad Sci B46* **6**, 745-752 (1980).

5. Subramanian S, Manoharan A, Ramaiah K, Das P. Rates of acquisition and loss of Wuchereria bancrofti infection in Culex quinquefasciatus. *Am J Trop Med Hyg* **51**, 244-249 (1994).

6. Garms R. Observations on filarial infections and parous rates of anthropophilic blackflies in Guatemala, with reference to the transmission of Onchocerca volvulus. *Tropenmed Parasitol* **26**, 169-182 (1975).

7. Ochoa A. JO. Studies on the anthropophilic blackfly species in Guatemala, with special reference to the transmission of onchocerciasis in the southeastern endemic area. *Medical Entomology and Zoology* **33**, 129-138 (1982).

8. Basanez MG, *et al.* Density-Dependent Processes in the Transmission of Human Onchocerciasis - Relationship between the Numbers of Microfilariae Ingested and Successful Larval Development in the Simuliid Vector. *Parasitology* **110**, 409-427 (1995).

9. Basanez MG, Boussinesq M. Population biology of human onchocerciasis. *Philos T Roy Soc B* **354**, 809-826 (1999).

10. Basanez MG, Collins RC, Porter CH, Little MP, Brandling-Bennett D. Transmission intensity and the patterns of Onchocerca volvulus infection in human communities. *Am J Trop Med Hyg* **67**, 669-679 (2002).

11. Filipe JAN, *et al.* Human infection patterns and heterogeneous exposure in river blindness. *P Natl Acad Sci USA* **102**, 15265-15270 (2005).

12. Basanez MG, Razali K, Renz A, Kelly D. Density-dependent host choice by disease vectors: epidemiological implications of the ideal free distribution. *T Roy Soc Trop Med H* **101**, 256-269 (2007).

13. Norman RA, *et al*. EPIFIL: The development of an age-structured model for describing the transmission dynamics and control of lymphatic filariasis. *Epidemiol Infect* **124**, 529-541 (2000).

14. Hairston NG, de Meillon B. On the inefficiency of transmission of Wuchereria bancrofti from mosquito to human host. *Bulletin of the World Health Organization* **38**, 935 (1968).

15. Duke BOL. Observations and Reflections on the Immature Stages of Onchocerca-Volvulus in the Human Host. *Ann Trop Med Parasit* **85**, 103-110 (1991).

16. Ho BC, Ewert A. Experimental transmission of filarial larvae in relation to feeding behaviour of the mosquito vectors. *T Roy Soc Trop Med H* **61**, 663-666 (1967).

17. Duerr H, Dietz K, Schulz-Key H, Büttner D, Eichner M. Density-dependent parasite establishment suggests infection-associated immunosuppression as an important mechanism for parasite density regulation in onchocerciasis. *T Roy Soc Trop Med H* **97**, 242-250 (2003).

18. Duerr HP, Dietz K, Eichner M. Determinants of the eradicability of filarial infections: a conceptual approach. *Trends Parasitol* **21**, 88-96 (2005).

19. Vanamail P, Subramanian S, Das PK, Pani SP, Rajagopalan PK. Estimation of Fecundic Life-Span of Wuchereria-Bancrofti from Longitudinal-Study of Human Infection in an Endemic Area of Pondicherry (South-India). *Indian J Med Res-A* **91**, 293-297 (1990).

20. Evans DB, Gelband H, Vlassoff C. Social and Economic-Factors and the Control of Lymphatic Filariasis - a Review. *Acta Trop* **53**, 1-26 (1993).

21. Ottesen EA, Ramachandran CP. Lymphatic Filariasis Infection and Disease - Control Strategies. *Parasitol Today* **11**, 129-131 (1995).

22. Vanamail P, Ramaiah KD, Pani SP, Das PK, Grenfell BT, Bundy DAP. Estimation of the fecund life span of Wuchereria bancrofti in an endemic area. *T Roy Soc Trop Med H* **90**, 119-121 (1996).

23. Scott AL. Lymphatic-dwelling filariae. *Lymphatic filariasis*. World Scientific (2000).

24. Duerr HP, Eichner M. Epidemiology and control of onchocerciasis: The threshold biting rate of savannah onchocerciasis in Africa. *Int J Parasitol* **40**, 641-650 (2010).

25. Subramanian S, Pani SP, Das PK, Rajagopalan PK. Bancroftian Filariasis in Pondicherry, South-India .2. Epidemiological Evaluation of the Effect of Vector Control. *Epidemiol Infect* **103**, 693-702 (1989).

26. Das P, *et al.* Bancroftian filariasis in Pondicherry, south India–epidemiological impact of recovery of the vector population. *Epidemiol Infect* **108**, 483-493 (1992).

27. Subramanian S, Krishnamoorthy K, Ramaiah KD, Habbema JDF, Das PK, Plaisier AP. The relationship between microfilarial load in the human host and uptake and development of Wuchereria bancrofti microfilariae by Culex quinquefasciatus: a study under natural conditions. *Parasitology* **116**, 243-255 (1998).

28. Grillet ME, *et al.* Vector competence of Simulium oyapockense s.l. and S. incrustatum for Onchocerca volvulus: Implications for ivermectin-based control in the Amazonian focus of human onchocerciasis, a multi-vector-host system. *Acta Trop* **107**, 80-89 (2008).

29. Soumbey-Alley E, *et al.* Uptake of Onchocerca volvulus (Nematoda: Onchocercidae) by Simulium (Diptera: Simuliidae) is not strongly dependent on the density of skin microfilariae in the human host. *J Med Entomol* **41**, 83-94 (2004).

30. Basanez MG, Townson H, Williams JR, Frontado H, Villamizar NJ, Anderson RM. Density-dependent processes in the transmission of human onchocerciasis: Relationship between microfilarial intake and mortality of the simuliid vector. *Parasitology* **113**, 331-355 (1996).

31. Richards FO, *et al.* Epidemiological and Entomological Evaluations after Six Years or More of Mass Drug Administration for Lymphatic Filariasis Elimination in Nigeria. *PLoS Negl Trop Dis* **5**, e1346 (2011).

32. Michael E, *et al.* Mathematical modelling and the control of lymphatic filariasis. *Lancet Infect Dis* **4**, 223-234 (2004).
